# Quantification of Internal Stress-Strain Fields in Human Tendon: Unraveling the Mechanisms that Underlie Regional Tendon Adaptations and Mal-Adaptations to Mechanical Loading and the Effectiveness of Therapeutic Eccentric Exercise

**DOI:** 10.3389/fphys.2017.00091

**Published:** 2017-02-28

**Authors:** Constantinos N. Maganaris, Panagiotis Chatzistergos, Neil D. Reeves, Marco V. Narici

**Affiliations:** ^1^School of Sport and Exercise Sciences, Liverpool John Moores UniversityLiverpool, UK; ^2^Faculty of Health Sciences, Staffordshire UniversityStoke-on-Trent, UK; ^3^School of Healthcare Science, Manchester Metropolitan UniversityManchester, UK; ^4^Faculty of Medicine and Health Sciences, School of Medicine, University of NottinghamDerby, UK

**Keywords:** Finite element modeling, tendon, eccentric exercise, tendinopathy, mechanical properties, plasticity

## Abstract

By virtue of their anatomical location between muscles and bones, tendons make it possible to transform contractile force to joint rotation and locomotion. However, tendons do not behave as rigid links, but exhibit viscoelastic tensile properties, thereby affecting the length and contractile force in the in-series muscle, but also storing and releasing elastic stain energy as some tendons are stretched and recoiled in a cyclic manner during locomotion. In the late 90s, advancements were made in the application of ultrasound scanning that allowed quantifying the tensile deformability and mechanical properties of human tendons *in vivo*. Since then, the main principles of the ultrasound-based method have been applied by numerous research groups throughout the world and showed that tendons increase their tensile stiffness in response to exercise training and chronic mechanical loading, in general, by increasing their size and improving their intrinsic material. It is often assumed that these changes occur homogenously, in the entire body of the tendon, but recent findings indicate that the adaptations may in fact take place in some but not all tendon regions. The present review focuses on these regional adaptability features and highlights two paradigms where they are particularly evident: (a) Chronic mechanical loading in healthy tendons, and (b) tendinopathy. In the former loading paradigm, local tendon adaptations indicate that certain regions may “see,” and therefore adapt to, increased levels of stress. In the latter paradigm, local pathological features indicate that certain tendon regions may be “stress-shielded” and degenerate over time. Eccentric exercise protocols have successfully been used in the management of tendinopathy, without much sound understanding of the mechanisms underpinning their effectiveness. For insertional tendinopathy, in particular, it is possible that the effectiveness of a loading/rehabilitation protocol depends on the topography of the stress created by the exercise and is not only reliant upon the type of muscle contraction performed. To better understand the micromechanical behavior and regional adaptability/mal-adaptability of tendon tissue it is important to estimate its internal stress-strain fields. Recent relevant advancements in numerical techniques related to tendon loading are discussed.

## Introduction

The primary role of tendon is to act as a mechanical link and transmit the contractile force generated by the muscle to the skeleton to produce joint rotation and whole-body movement. Clearly, this role necessitates that the tendon is stiff, however, tendons also exhibit a viscoelastic behavior when stretched, which has implications for joint positioning control, joint moment and power generation and metabolic energy expenditure during terrestrial locomotion (for reviews see Zajac, 1989; Alexander, 1991; Magnusson et al., 2008). Importantly, tendons adapt to their mechanical environment by increasing or decreasing their stiffness depending on the requirement for increased or reduced use and safe function. This review paper presents evidence that tendons may adapt and mal-adapt regionally and not homogenously throughout their body, which presents new challenges for our understanding of how tendons transmit and respond to tensile forces. The paper is broadly split in three parts: Firstly, we present findings from *in vivo* studies showing that there are various possible mechanisms through which tendons may change their stiffness in response to alterations in chronic mechanical loading. We then examine two diverse paradigms in which region-specific effects are evident and stand in opposition to the simplistic approximation of a tendon and its muscle being always mechanically in-series with each other: (a) Healthy tendon adaptation to chronic mechanical loading, and (b) chronic tendon pathologies and their management with eccentric exercise. The final part of the review is dedicated in making the case for developing and validating new *in vivo*-measurement based tools for the quantification of local tendon stresses and strains to better understand the reasons underpinning regional adaptability and mal-adaptability in human tendon.

## Plasticity of human tendon to mechanical loading

Despite their poor vascularity, tendons are metabolically active structures and display remarkable plasticity when subjected to tensile loading (Magnusson et al., 2008; Arampatzis et al., 2009; Wiesinger et al., 2015). The hypothesized mechanism underpinning the adaptability of tendons and all load-bearing biological structures is *mechanotransduction*, i.e., the conversion of mechanical stimuli into a cascade of chemical events leading to the production of new load-bearing material (Chiquet et al., 2009). Observations made on both animals (Heinemeier et al., 2007) and humans (Miller et al., 2005) indicate that mechanical loading of a tendon triggers an acute increase in collagen expression and increased collagen protein synthesis as well as an increase of collagen protein degradation (Langberg et al., 1999). Indeed, there is a very fine balance between synthesis and degradation, which can be disrupted when the tendon receives too much or too little mechanical stimulation (Arnoczky et al., 2007).

Until recently, knowledge on tendon mechanical behavior could only be gained from *in vitro* material tests, where typically a piece of tendon gripped at both ends is pulled on by a motor, while recordings of the force applied and the deformation caused are made and plotted against each other to calculate slopes and areas (for reviews see Viidik, 1973; Butler et al., 1978; Ker, 1992). *In vitro* tendon testing protocols have been instrumental for the identification of tendon grafts suitable for surgical ligament reconstruction, but it is clear that lab tests do not represent accurately *in vivo* tendon loading conditions, e.g., as opposed to an isolated specimen, *in vivo* tendon is metabolically active when pulled on by the in-series contracting muscle, and joint shape and rotation can influence the direction of force acting on the tendon and the consequent deformation. Therefore, *in vitro* testing results should be treated with caution when used to infer *in vivo* function. Equally important is the obvious limitation that *in vitro* tensile protocols do not allow the implementation of longitudinal experimental designs.

**Figure 1:**
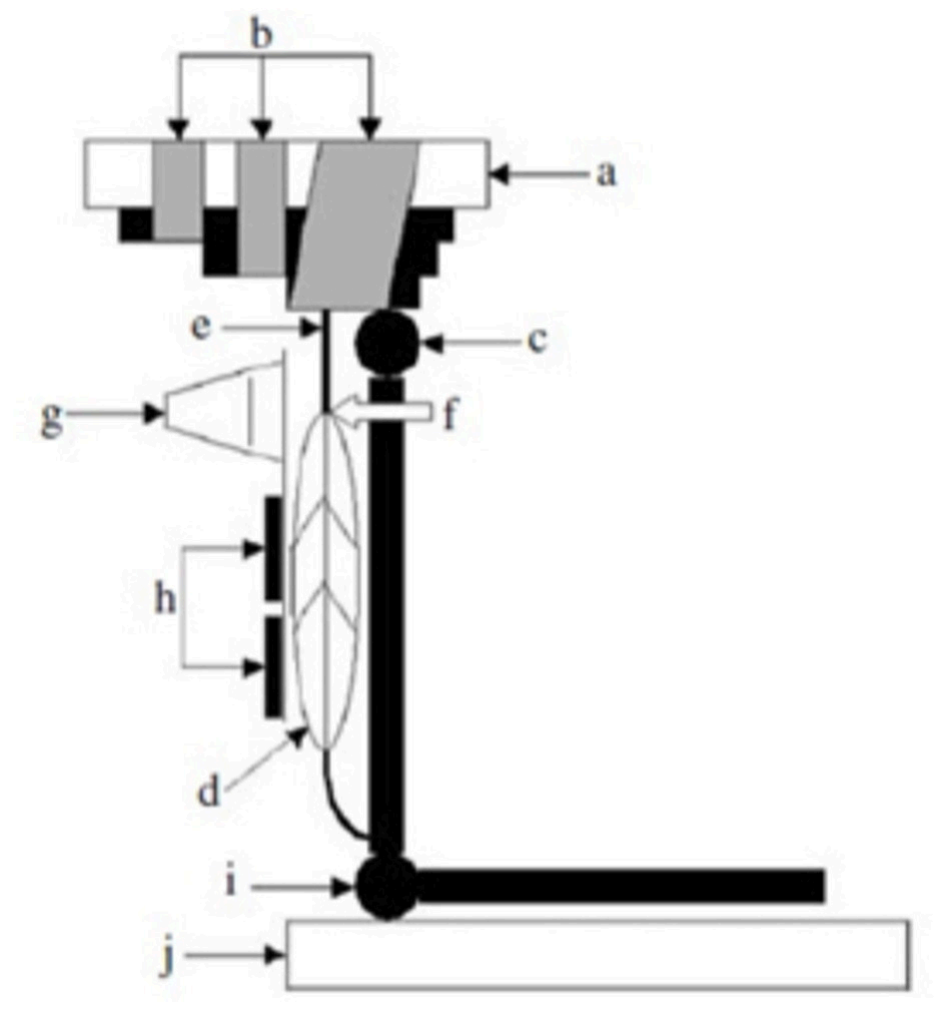
**Experimental set-up for the ***in vivo*** measurement of the human tibialis anterior (TA) tendon mechanical properties**. (a) dynamometer footplate, (b) velcro straps, (c) ankle joint, (d) tA muscle, (e) TA tendon, (f) myotendinous junction, (g) ultrasound probe, (h) percutaneous stimulating electrodes for activating the TA muscle, bypassing voluntary co-contraction of antagonist muscles, (i) knee joint, (j) knee mechanical stop. From Maganaris and Paul (2000).

A methodology for studying *in vivo* the mechanical behavior of human tendons overcoming the above limitations was developed in the late 90s (Maganaris and Paul, 1999; Figure 1). This methodology is based on real-time, sagittal-plane ultrasound scanning of a reference point along the tendon during a ramp intensity isometric contraction of the in-series muscle, producing tendon forces that can be quantified from dynamometric measurements of joint moment and the moment arm length of the tendon about the joint. The tendon forces corresponding to the tendon deformations quantified from the recorded scans can then be reduced to stress values by normalization to the Cross Sectional Area (CSA) of the tendon, measured from axial-plane Magnetic Resonance Imaging (MRI) or ultrasound scans. Tendon stiffness and Young's modulus values can be calculated from the slopes of the force-deformation and stress-strain plots.

Despite inter-study differences in the measurement of tendon deformations (e.g., including the aponeurosis or scanning the free tendon only) and quantification of tendon forces (e.g., correcting for the moment of the antagonist muscles or assuming zero co-activation), the main principles of the above methodology have been implemented in numerous studies over the last 20 years to study the adaptability of human tendons to exercise training and mechanical loading in general (for reviews see Magnusson et al., 2008; Arampatzis et al., 2009; Seynnes et al., 2015; Wiesinger et al., 2015). The findings reviewed show convincingly that human tendons respond to the application of increased mechanical loading by becoming stiffer. The mechanisms underpinning this adaptation include tendon hypertrophy (increased tendon CSA) and improvement in tendon material (increased Young's modulus). Importantly, the findings differ between studies, with some studies reporting adaptations in tendon size but not material (Rosager et al., 2002; Couppé et al., 2008), others in tendon material but not size (Kubo et al., 2001; Reeves et al., 2003; Malliaras et al., 2013a), and yet some others reporting adaptations in both tendon size and material (Arampatzis et al., 2007a,b; Kongsgaard et al., 2007; Seynnes et al., 2009; Stenroth et al., 2016). To further explore these diverse adaptive responses, we should look in more detail at the loading characteristics implemented in relation to the anatomical location and function of the tendons studied and the experimental designs opted for by different authors.

Cross-sectional experimental designs have often been adopted for the following purposes: (A) For comparisons of human tendons subjected to different habitual loads due to their anatomical location, e.g., the tibialis anterior tendon, a major ankle dorsiflexor tendon which does not carry high forces during locomotion vs. the gastrocnemius tendon, a major ankle plantarflexor tendon which acts like a spring during locomotion and can carry loads approximating the tendon's ultimate tensile stress (Maganaris, 2002; Maganaris and Paul, 2002), or to compare tendons between limbs with asymmetry in muscle strength (Couppé et al., 2008); (B) For comparisons of lower-limb tendons in humans with different body mass but similar habitual activities (Wiesinger et al., 2016); (C) For comparisons of tendons in different types of athletes vs. sedentary individuals (Kubo et al., 2000a,b; Magnusson and Kjaer, 2003; Arampatzis et al., 2007b; Stenroth et al., 2016). Studies adopting the experimental designs (A) and (B) above have shown that the tendon's Young's modulus does not correspond to its anatomical location or habitual loading, and the ratio of tendon CSA to body mass is rather constant. These findings agree with animal data (Pollock and Shadwick, 1994) and support the notion that adjustments in tendon stiffness to accommodate changes in physiological loading are accomplished by adding or removing material rather than altering the material's intrinsic properties. Findings from experimental model (C) type studies, however, have shown that alterations in tendon material may occur and account fully for or contribute to the increased tendon stiffness in response to loading. Interestingly, exercise training intervention studies also report improvements in tendon Young's modulus. In combination with the results from study types (A) and (B), the finding of a higher Young's modulus in athletes and people engaged in exercise training interventions indicates that stiffening of the tendon through alteration of its material requires “supra-physiological” loading features, e.g., in terms of loading magnitude, frequency and/or duration. Once this rapid adaptation occurs and the exercise becomes habitual daily activity, alterations in tendon size might mediate any further changes in tendon stiffness, as suggested by the findings in type (A) and (B) studies above.

## Regional size adaptations to loading in healthy tendon

Increases in tendon CSA in response to chronic mechanical loading are typically inferred as tendon hypertrophy, i.e., newly synthesized load-bearing material that will decrease the overall stress applied. Although not much is known about the actual composition of the new material, microdialysis-based findings show increased levels of collagen synthesis markers in human peritendinous tissue in response to acute and long-term exercise (Langberg et al., 1999, 2001). However, recent findings based on the pattern of incorporation in the tendon of carbon-14 generated by nuclear bomb tests in the 50s and 60s, indicate that the core of the tendon remains largely inert in mature adults (Heinemeier et al., 2013). Taken together, these findings suggest that hypertrophy in response to exercise training in mature humans may preferentially occur in the periphery of the tendon. However, it cannot be excluded that some tendon size increase reflects an increased water concentration in the area through the augmented proteoglycan content stated above. From a mechanical point of view this tendon size increase is rather “non-functional” and would have little bearing on the maximum load that the tendon can carry before it raptures in tension.

**Figure 2:**
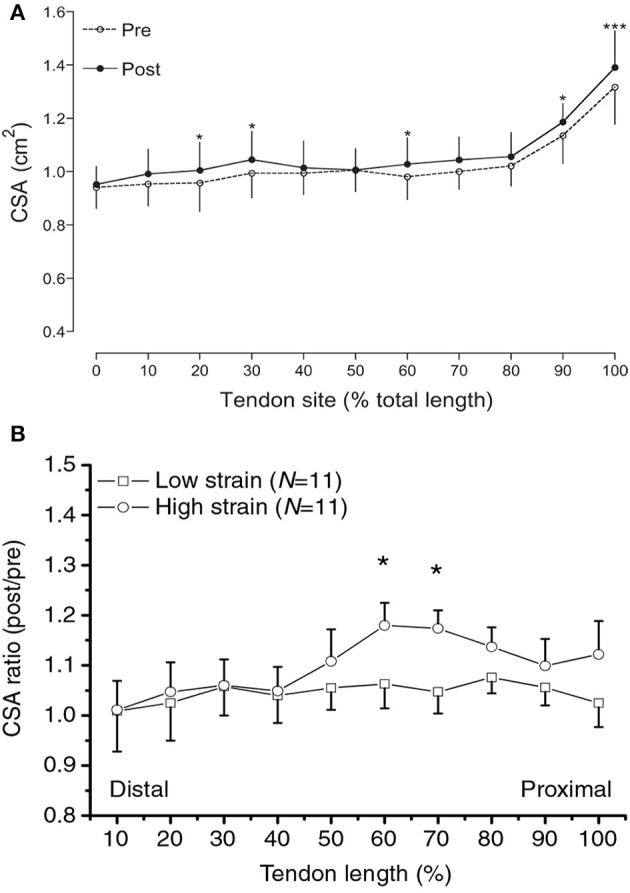
**(A)** Regional increases in patellar tendon CSA along the tendon's length in response to 16 weeks of strength training in young adults. Reproduced from Seynnes et al. (2009). **(B)** Regional increases in Achilles tendon CSA along the tendon's length in response to high-strain vs. low-strain contractions. Reproduced with permission from Arampatzis et al. (2007a). Asterisks indicate differences in tendon size between conditions.

Regional tendon plasticity is a feature also seen when considering the CSA of the tendon along its whole length, from origin to insertion. Although recent advancements in 3D ultrasound scanning applications allow a detailed mapping of tendon CSA along the entire tendon length (Obst et al., 2014a,b), typically, tendon CSA has been measured in one to three axial-plane scans (Kubo et al., 2001; Reeves et al., 2003; Malliaras et al., 2013a; Couppé et al., 2014) and an average value has been used to infer adaptation, or lack of, in tendon size. A lack of tendon CSA alterations to loading has often been reported following this measurement approach (Kubo et al., 2001; Reeves et al., 2003; Malliaras et al., 2013a). In contrast, studies in which the CSA of the tendon has been quantified in a large number of scans along the entire tendon (Figure 2) show that there are tendon regions which preferentially hypertrophy, toward the osteo-tendinous junction or elsewhere in the main body of the tendon, while others remain unchanged (Magnusson and Kjaer, 2003; Arampatzis et al., 2007a; Seynnes et al., 2009). However, there are also studies that have documented regional tendon hypertrophy with only three scans along the tendon (Kongsgaard et al., 2007; Couppé et al., 2008). The finding of regional plasticity raises two important issues: (1) It makes doubtful the conclusion of lack of tendon hypertrophy in studies with tendon CSA data from few only scans; hypertrophy may have actually occurred at a different tendon level than that scanned but gone undetected. (2) It highlights that the model of a spring (tendon) loaded by a mass (muscle force) may be overly simplistic when considering the mechanisms of contractile force transmission to the skeleton. This model assumes that every single point along the tendon in series with the muscle “sees” the same force and a homogenous adaptation throughout the tendon's body should therefore be expected if there are no differences in mechanotransduction sensitivity between tendon regions. Having a means available to quantify local stresses would be a key step in understanding the mechanism of local tendon adaptations.

## Regional tendon mal-adaptations in tendinopathy

Despite the remarkable ability of tendon to withstand high tensile loads, tendon overuse injuries often occur with the application of relatively small, but repetitive loads. Tendinopathy is a common disorder characterized by pain during activity, localized tenderness upon palpation, swelling of the tendon, tissue degeneration and impaired performance (Maganaris et al., 2004; Maffulli et al., 2005; Arnoczky et al., 2007; Magnusson et al., 2010; Malliaras et al., 2013a; Pearson and Hussain, 2014). Recent ultrasound-based measurements have shown that tendons with tendinopathy have a reduced stiffness and Young's modulus but a larger “non-functional” CSA (see also above) than healthy tendons (Arya and Kulig, 2010). It is uncertain whether these alterations result from, or in fact precede the development of symptomatic tendinopathy. If, however, the latter is the case then a relatively simple scanning-based procedure could be used to predict the risk of developing tendinopathy. Two clinically important observations relating to chronic tendon injuries are noteworthy: (1) Certain tendons are affected more often than others, and (2) the anatomical location of the lesion is not consistent across tendons. The Achilles and patellar tendons are amongst the most commonly affected tendons by tendinopathy (Maganaris et al., 2004; Maffulli et al., 2005; Malliaras et al., 2013a; Pearson and Hussain, 2014). The patellar tendon is almost exclusively affected by insertional tendinopathy, i.e., tendinopathy in the osteo-tendinous junction region, usually in the proximal end of the tendon (Maffulli et al., 2005; Maganaris et al., 2004; Malliaras et al., 2013a; Pearson and Hussain, 2014). The Achilles tendon, on the other hand, is affected by both insertional tendinopathy and by tendinopathy of the main body or of the structures surrounding the tendon (Maganaris et al., 2004; Maffulli et al., 2005; Malliaras et al., 2013a).

A deep understanding of the etiology of tendinopathy is of paramount importance for preventing it as well as for treating it. Despite the progress achieved over the last decades there is still great uncertainty over the factors causing this pathology and controlling its progression (Maffulli et al., 2005; Arnoczky et al., 2007; Magnusson et al., 2010). A widely-accepted concept for explaining tendinopathy development is the generation of excessive tensile loads within the tendon over time. If a rapid increase in the magnitude, duration or frequency of loading occurs, tensile strength might be exceeded locally causing a micro-injury. The mechanism that causes the progression of this micro-injury is not yet definitively known, but is generally believed that repetitive overloading of the tendon may overwhelm the tissue's healing capacity causing a more severe injury. This concept identifies excessive tensile load to be the key factor in tendinopathy development (Leadbetter, 1992).

However, a number of clinical and biomechanical findings at different hierarchical levels indicate that there is more into the etiology of tendinopathy than just excessive tensile load. From a purely mechanical point of view, the tendon is “over-engineered” compared to the attached muscle. Indeed, experimental models of muscle-tendon unit rupture in tension have shown that failure occurs within the muscle—not in the tendon (Almekinders and Gilbert, 1986; Garrett, 1990). Another interesting observation from histopathology findings using material excised during surgery in tendinopathy patients (Griffiths and Selesnick, 1998) and cadaveric material (Rufai et al., 1995) is that in insertional tendinopathy the lesion appears systematically at the joint side of the enthesis. This anatomical area has a complex internal structure with a cartilagenous transition zone from tendon to bone tissue, which is more pronounced on the joint side of the tendon (Benjamin et al., 1986). Histological findings have shown that fibro-cartilagenous metaplasia can occur in the tendon's enthesis as an adaptive response to compression (Vogel et al., 1993). Excessive tendon compressive loading, e.g., caused by frequent operation of the joint at end range of movement positions under high muscle forces, could locally reduce the tendon's tensile strength making it more prone to injury. Moreover, a study in which patellar tendons in fresh frozen knees were instrumented with strain gauges directly showed that the proximal enthesis region, which typically develops tendinopathy, is subjected to very low longitudinal strains, indicative of low tensile stresses (Almekinders et al., 2002). This lack of adequate local stress, often referred to as “stress-shielding” (for a review see Maganaris et al., 2004) could lead to tensile weakening and degeneration over time. Recent studies using ultrasound speckle tracking have provided more direct *in vivo* evidence that human tendons may undergo non-uniform displacements during passive or active force application, with deeper tissue layers deforming more than superficial ones (Korstanje et al., 2010; Kim et al., 2011; Slane and Thelen, 2015). Again, quantification of local stress characteristics, within the pathological area and/or tendon regions displaying non-uniform deformations on stretching, would be pivotal in unraveling the mechanisms involved and establishing if tendinopathy is more prominently linked with the development of (1) strong tensile stress concentration, (2) strong compressive stress concentration, or (3) stress-shielding.

## Eccentric exercise and tendinopathy

Despite the morbidity degree associated with chronic tendinopathy and the extent of knowledge in certain areas of medical treatment, both clinicians and scientists involved in tendon biology/biomechanics acknowledge that there is a surprising lack of scientific rationale for justifying why some treatments are more effective than others (Maffulli et al., 2005; Kjaer and Heinemeier, 2014). Over the past decade, eccentric exercise training has successfully been used in the clinical management of tendinopathy (for a review see Frizziero et al., 2014). While the clinical evidence for the beneficial role of eccentric exercises is overwhelming, the mechanism underpinning the effectiveness of eccentric contractions has not been documented, nor is there any evidence that this type of muscle contraction is in fact the most effective one (for a review see Malliaras et al., 2013b). It is possible that eccentric contractions, if performed with maximal volition, are more effective than concentric contractions because they subject the in-series tendon to a greater tensile force. Indeed, experiments on healthy human tendons have shown that exercise training involving higher intensity contractions result in greater tendon stiffness changes than lower intensity contractions (Arampatzis et al., 2007a; Malliaras et al., 2013a). For the same muscle force, however, the direction of muscle deformation in an exercise protocol should have no impact on the magnitude of tendon mechanical and morphological changes, as the tendon would “see” the same load (muscle force) and therefore undergo the same strain. It has also been shown that eccentric contractions generate greater changes in tendon blood circulation than concentric contractions (Kubo, 2015), but this would again be linked to the greater muscle contractile force applied and transferred along the tendon when a muscle is forcefully lengthened. Eccentric and concentric contractions elicit similar levels of fibroblast activation (Heinemeier et al., 2007). However, eccentric contractions generate high-frequency oscillations in the tendon (Rees et al., 2008), a form of loading which has recently been shown to stimulate collagen synthesis in intact animal tendons (Thompson et al., 2015). In line with the notion that force oscillations underpin the effectiveness of eccentric exercise in tendinopathy is the finding of a more recent study showing healthy human tendon hypertrophy in response to vibration training (Rieder et al., 2016). Eccentric exercise has also been shown to decrease acutely the thickness of tendon more than concentric exercise, indicating increased water outflow due to increased compressive forces between collagen fibers, potentially caused by a less uniform distribution of contractile stresses generated by activation of fewer motor units (Grigg et al., 2009). Water mobilization has been shown to stimulate the production of factors such as cyclooxygenase II and matrix metalloproteinases in healthy animal tendon cells (Archambault et al., 2002) and more work is required to better elucidate the effect of these genes up-regulation with fluid flow on tissue remodeling in tendon pathologies. One other possibility is that eccentric contractions are effective, especially in insertional tendinopathy, because they allow the pathologic “stress-shielded” area to “see,” and therefore adapt to, some of the stress carried along the main body of the tendon as the muscle-tendon unit is lengthened slowly throughout the whole range of joint movement. In fact, it has been shown that resistance training with high intensity contractions applied slowly throughout the physiological range of joint movement is equally effective with eccentric training in terms of physical activity improvement and pain reduction in patients with patellar tendinopathy (Beyer et al., 2015). Thus, it seems possible that the effectiveness of an exercise regimen depends on the loading “seen” by the pathological area, which is a function of muscle contraction intensity, muscle contraction velocity and range of joint movement covered, and not solely contraction type. Somewhat in contrast to the above notion is the conclusion of a recent systematic review that functional improvements and pain reduction in response to eccentric exercise in patients with tendinopathy involves mechanisms distinct from local structural adaptations in the tendon (Drew et al., 2014). However, the above review was predominantly based on patients with mid-portion Achilles tendinopathy, a pathology with potentially different pathomechanical basis than insertional tendinopathy. Interestingly though, eccentric exercise is effective for managing mid-portion tendinopathy too (Habets and van Cingel, 2015), although it has recently been shown that heavy and slow resistance training is equally effective in terms of clinical outcomes (Beyer et al., 2015). For insertional tendinopathy at least, information of local stresses elicited in the pathological tendon region during different types of contractions and joint manipulations seems pivotal in establishing why some protocols are more effective than others, thereby allowing the design of effective, patient-specific rehabilitation regimens.

## Quantification of internal stress-strain fields in tendon

From the above analysis it becomes apparent that the mechanical approximation of tendon as a single, homogenous force-transmitting structure prevents us from understanding the mechanisms underpinning tendon adaptations and mal-adaptations. A fundamental question that remains unanswered is whether tensile forces are transmitted evenly through the tendon resulting in homogeneous stress distribution and whether any non-homogenous behavior has consequences for the plasticity that the tendon exhibits in response to mechanical loading, pathology and exercise rehabilitation.

Understanding the micromechanical behavior of tendon tissue and estimating its internal stress-strain fields is fundamental for understanding how tendons adapt to loading and pathology. *In vivo* tensile tendon forces can be directly quantified using implantable sensors such as buckle force transducers, load cells, liquid metal strain gauges and fiber optic transducers (Bey and Derwin, 2012). More specifically, buckle transducers and fiber optic transducers have been used to measure *in vivo* forces (Gregor et al., 1987; Komi, 1990; Fukashiro et al., 1995; Finni et al., 1998, 2000) in the human Achilles and patellar tendons (Finni et al., 1998). Even though these studies have given invaluable new insight into the magnitude of *in vivo* tensile loading experienced by tendons during activities of daily living, their use has been significantly limited mainly due to their invasive nature and concerns about reliability (Fleming et al., 2000; Bey and Derwin, 2012). Moreover, because these sensors are designed to stay inside the human body only for small periods of time (i.e., several hours) they are not suitable for longitudinal studies where changes in tendon biomechanics have to be assessed and followed-up over long periods of time (Bey and Derwin, 2012). A final significant limitation of all implantable sensors is their inability to offer full-field measurements of stress or strain.

The only technique that enables non-invasive estimation of full fields of internal tendon forces/stresses and deformations/strains is Finite Element (FE) modeling. Even though FE modeling has significantly enhanced practice in almost every engineering application, this success has not yet been extended outside traditional engineering (Miller and Lu, 2013). In the case of tendon biomechanics, extracting clinically meaningful data capable of enhancing clinical practice has been proven exceptionally challenging. This is mainly due to the amount of *in vivo* data that is needed to support the design of FE models (e.g., in terms of geometry, material properties etc.) and their validation (Viceconti et al., 2005).

## Reconstruction of geometry

Investigating the internal stress-strain fields of tendons requires 3D models with realistic and accurately reconstructed geometry. The importance of subject-specific geometry was recently highlighted by Shim et al. (2014) who analyzed the numerically predicted stress-strain fields of 10 tendons using generic or subject-specific geometry. Their results revealed that the location where the strongest stresses were observed (i.e., the locations more likely to be overloaded and therefore injured) was significantly influenced by initial geometry (Shim et al., 2014). This finding indicates that accurate reconstruction of subject-specific tendon geometry is of paramount importance for assessing regional differences in internal stresses-strains and for studying their effect on tendon biomechanics.

In the study above, subject-specific geometry was reconstructed based on ultrasound imaging (Shim et al., 2014). In contrast to CT and MRI, ultrasound is relatively easy to use, safe (both for the patient and the operator) and characterized by low cost and easy access. On the other hand, the disadvantages of ultrasound include relatively limited field of view, low contrast compared to MRI/CT, and the fact that the quality of imaging is strongly user-dependent. Besides its limitations, ultrasound imaging is a very good candidate for applications focused on tissues that are close to skin (Kim et al., 2011; Shim et al., 2014; Obst et al., 2014a,b; Behforootan et al., 2017). Moreover, concerns about user dependency could be addressed by automated movement of the scanning probe (e.g., Docking and Cook, 2016).

Despite its technical challenges, 3D imaging of tendons is a very useful tool for investigating tendon biomechanics (Obst et al., 2014a,b). Indeed, being able to reconstruct the 3D shape of tendons under different *in vivo* loading conditions enables the quantification of the resultant local longitudinal and transverse deformations. This can shed light on possible regional phenomena (e.g., relative sliding between neighboring parts of the tendon, localized stiffening etc.) that cannot be studied using more global approaches, where imaging is only used to measure overall tendon elongation. However, in exceptional cases and with the prerequisite that the simulated scenario doesn't involve significant out-of-plane forces or displacements, insight into internal stress-strain fields can also be gained from the use of 2D models. Two-dimensional models are substantially simpler to design compared to 3D models and their computational cost can be significantly lower. However, their clinical relevance and range of applications is also significantly restricted by the aforementioned limitation in terms of out of plane forces and displacements. An interesting application of a 2D model of the patellar tendon was presented by Lavagnino et al. (2008). A 2D FE model of the patella and the patellar tendon was designed based on lateral radiographs of a human knee and assuming that the patellar tendon is a homogenous and isotropic hyperelastic material. This model was used to calculate the strains that are developed within the tendon during jump landing and compare them against *in vitro* observations. The comparison revealed a correlation between the areas of maximum principal strain and the respective areas where collagen fascicle disruption was observed *in vitro* (Lavagnino et al., 2008). Among others this study highlights the potential to correlate numerical results (i.e., in this case location of maximum strain) with experimentally observed micro-damage or trauma. However, in interpreting and assessing the clinical relevance of these results, certain limitations regarding the simulation of tendon mechanical behavior need to be considered. More specifically, the fact that patellar tendon was simulated as a homogenous isotropic material means that the location where maximum internal strain is calculated is dictated only by geometry and loading. Indeed, localized stiffening/softening could significantly change internal strains and the location where strains are maximized. Therefore, it becomes, clear that the aforementioned correlation actually indicates a link between non-uniformities in internal loading and trauma and also highlights the need for more realistic simulation of tendon mechanical properties including the simulation of possible localized softening/stiffening.

## Calculation of material properties

The most commonly used method for the quantitative non-invasive assessment of soft tissue material properties is FE inverse engineering. According to this technique a FE model that simulates a specific *in vivo* test is designed and used in an optimization-based process. In each step of this highly iterative process, numerical estimations in terms of tissue strain or stress are compared against experimental results and the material coefficients of the FE model are updated to minimize the difference between numerical simulation and *in vivo* experiment. Provided that convergence to the global optimum of this minimization problem is achieved, inverse engineering ensures maximum agreement between simulation and physical world. However, this agreement is limited to the specific aspects of *in vivo* behavior that are quantified by the *in vivo* data fed into this process. This technique has been used to calculate *in vivo* mechanical properties, including hyperelastic and viscoelastic material coefficients, in a wide range of tissues with different mechanical characteristics (Tang et al., 2011; Isvilanonda et al., 2012; Hassaballah et al., 2013; Petre et al., 2013; Chatzistergos et al., 2015). In the case of tendons, tensile force-deformation data were used to inverse engineer three material coefficients that are needed to describe tendon hyperelasticity according to the Mooney-Rivlin model (1st order; Tang et al., 2011). The number of material coefficients that can be calculated with this approach is strongly dependent on the nature of *in vivo* data. In most cases though, the only information available on tissue mechanical behavior is a load-deformation curve that describes the macroscopic behavior of the entire tissue under tension or compression. Acquiring a more detailed mapping of material properties requires more in-depth information on the force-deformation behavior of different regions of the tissue (Petre et al., 2013).

Even though the measurement of tendon overall longitudinal deformation by tracking a single reference point (Maganaris and Paul, 1999) is well established and has been widely applied (for reviews see Magnusson et al., 2008; Arampatzis et al., 2009; Seynnes et al., 2015; Wiesinger et al., 2015), this method does not allow quantification of longitudinal deformations in localized tendon areas. However, recent developments in ultrasound speckle tracking algorithms have made it possible to quantify human tendon regional longitudinal displacements in tension and map *in vivo* deformability patterns in 2D scans (Kim et al., 2011; Arndt et al., 2012; Slane and Thelen, 2015). Advancements have also been in the measurement of human tendon CSA. Transverse deformations of the human Achilles tendon under tension, created by voluntary isometric plantarflexion, were recently measured *in vivo* using ultrasound imaging (Obst et al., 2014b). The structural measurements and the measurement of transverse deformations were performed along the length of the tendons enabling the reconstruction of their initial and deformed 3D shapes. From these data the transverse rotations and strains of Achilles tendons along their length were mapped and it was noted that both deformations were maximized in the mid-portion of the free Achilles tendon. In addition to the clear implications of this finding for understanding injury mechanisms, quantification of 3D deformations can be implemented to better understand the relation between regional structural adaptations and stresses in the tendon.

**Figure 3:**
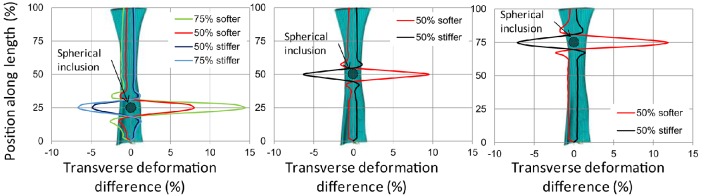
**Difference (%) between the transverse deformations at 10% elongation of a homogenous tendon and tendons with localized softening/stiffening at 25% of tendon length (left)** 50% of tendon length **(center)** and 75% of tendon length **(right)**. The position of localized softening/stiffening (i.e., spherical inclusion) is marked with a circle. From Chatzistergos et al. (2016).

In this direction, we performed a pilot numerical study to assess the effect of localized softening or stiffening on the transverse deformations of a tendon under tension (Chatzistergos et al., 2016). For this purpose, a 3D FE model of Achilles tendon with homogenous material properties was designed from medical imaging. This model was subjected to 10% elongation and its transverse deformations were mapped along its length. Localized softening or stiffening was simulated by introducing spherical inclusions with a material that is either softer or stiffer than the rest of the tendon. Comparing the transverse deformations between the FE models with uniform and non-uniform material properties indicated that localized softening or stiffening causes localized increase or decrease in transverse deformations, respectively. Moreover, the position where differences in terms of transverse deformations were maximized also marked the position of localized softening/stiffening (Figure 3), indicating that transverse deformations of a tendon under tension could enable the detection of localized softening/stiffening. Based on these pilot numerical results we concluded that techniques for the accurate measurement of *in vivo* transverse deformations could potentially be used to support a quantitative assessment of regional differences in tendon mechanical properties (Chatzistergos et al., 2016).

In terms of mechanical properties, the FE simulation of tendon biomechanics is dominated by the assumption of homogeneity. This simplification mainly stems from the difficulty to quantitatively assess possible regional differences in the mechanical properties of tendons (Lavagnino et al., 2008). The need to move away from the assumption of tendon mechanical homogeneity and toward techniques that enable assessing possible regional differences in tendon mechanical properties is highlighted in recent studies using shear wave elastography (DeWall et al., 2014; Aubry et al., 2015). More specifically, DeWall et al. (2014) observed a significant decrease in shear wave speeds along the length of healthy Achilles tendons from the distal, bonny end of the tendon toward the musculotendinous junction (DeWall et al., 2014). Even though the exact relationship between shear wave speed and tissue mechanical properties is not yet fully understood, the aforementioned observation is a strong evidence for non-uniform mechanical properties in healthy tendons. In a similar study, shear wave speeds of Achilles tendons with tendinopathy were compared against healthy Achilles tendons (Aubry et al., 2015). Despite the fact that the two groups were not aged-matched and the effect of pathology could not be entirely separated from that of aging, a reduction in shear wave speed indicative of tissue softening was observed in tendinopathic tissue, which highlights the need for quantitative assessment of regional differences in the mechanical properties of tendons. However, certain limitations need to be considered in the interpretation of shear wave elastography data. One limitation is that shear wave speed is affected by the thickness of the tissue scanned (Brum et al., 2014), meaning that differences in tendon shear wave speed may not necessarily correspond to changes in tendon material stiffness. This limitation applies specifically to tendons because their average thickness is smaller than the wavelength of shear waves, leading to successive reflections at tendon boundaries and a guided propagation of shear waves along the length of the tendon (Brum et al., 2014). One other important consideration is that there is a saturation point in the maximum shear wave speed that can be recorded, dictated by the tracking capacity of the elastography scanner (DeWall et al., 2014). Clearly, beyond that point any changes in stiffness and other factors that affect shear wave speed cannot be assessed. Unfortunately, the shear wave tracking capacity of current elastography technology is rather limited, and for tendon the saturation point can be reached by a slight stretch, by means of joint rotation (DeWall et al., 2014), which is much smaller than the tendon deformation and the respective stiffness achieved when the muscle is active and pulls on the tendon. This means that the shear wave speeds recorded at tendon force and stiffness values relevant to *in vivo* functioning are underestimates of the true speed of propagation of the waves. Future elastography developments will hopefully yield tracking capacity speeds higher than the shear wave propagation speed in tendons when stretched by physiologically relevant forces.

Studying possible regional differences in the mechanical properties of tendons with tendinopathy can shed new light on both the etiology of tendinopathy and its impact on tendon biomechanics. Besides that, studying possible regional differences in the mechanical properties of both healthy tendons and tendons with tendinopathy is also very important for our understanding on the adaptive responses of tendon to loading. As mentioned earlier, longitudinal studies involving chronic exercise training have shown that chronic loading can cause localized changes in tendon CSA and changes to their macroscopic stiffness (Seynnes et al., 2009). Even though localized changes in structure is also an indicator for localized changes in mechanical properties, this possibility has not been yet investigated. Understanding the possible localized effect of different exercises on tendon mechanical properties can open the way for new personalized and targeted approaches to tendon rehabilitation. Based on the above we suggest that the development of techniques for the quantitative assessment and mapping of regional differences in tendon mechanical properties is a critical next step for enhancing our understanding of the pathomechanical features of tendon pathology and for improving the efficacy of rehabilitation. Current literature on novel applications of ultrasound imaging highlight shear wave elastography and the mapping of 3D deformations of tendons under tension as the two most promising techniques in this direction.

## Validation

Validation is the ultimate challenge for extracting clinically meaningful data from FE analyses. Direct validation involves the direct comparison between numerical and experimental results for scenarios that closely match the ones under study (Viceconti et al., 2005). The simplest approach for the validation of tendon FE models would be to compare numerically estimated macroscopic force-elongation graphs with *in vivo* measured ones. However, it must be emphasized that such graphs reflect the mechanical behavior of the entire tendon, thus they cannot be used to validate the models' ability to map internal stresses. This means that in order to validate the ability of FE models to map stress fields in tendons, numerically calculated stresses should be directly compared to *in vivo* measured ones. However, and as it was discussed at the beginning of this paragraph, internal tendon stresses cannot be directly measured non-invasively. This makes the direct validation of stress field calculations practically impossible and highlights the need for alternative approaches to validation.

In contrast to tendon stresses, tendon strains can indeed be directly measured using medical imaging modalities such as such as ultrasound (Kim et al., 2011; Arndt et al., 2012; Obst et al., 2014b; Slane and Thelen, 2015). This makes measurements of *in vivo* 3D strains very useful for the validation of FE models too. Comparing the numerically calculated 3D strain fields to *in vivo* measured ones can offer a robust and in-depth validation of FE models that goes beyond validating just their macroscopic response to loading. Moreover, assuming that regional differences in material properties of tendon have been accurately assessed (e.g., using shear wave elastography etc.) and incorporated into FE modeling, *in vivo* measurements of the 3D strains of tendons could also be used to validate the ability of FE models to predict internal stress fields too.

To conclude, the combined use of non-invasive measurement techniques for *in vivo* tendon strains and FE modeling has the potential to significantly enhance our understanding of the mechanisms involved in tendon adaptability and mal-adaptability to mechanical loading and to inform the design of targeted rehabilitation exercises that are mechanistically and effectively addressing patient-specific tendon pathologies. Specific aspects of clinically relevant FE modeling that are challenging but important to address include (i) the non-invasive mechanical characterization without the assumption of homogeneity and (ii) direct in-depth validation.

## Author contributions

All authors listed, have made substantial, direct and intellectual contribution to the work, and approved it for publication.

### Conflict of interest statement

The authors declare that the research was conducted in the absence of any commercial or financial relationships that could be construed as a potential conflict of interest.
